# Composite Confirmed Disability Worsening/Progression Is a Useful Clinical Endpoint for Multiple Sclerosis Clinical Trials

**DOI:** 10.1212/WNL.0000000000213558

**Published:** 2025-04-21

**Authors:** Ludwig Kappos, Sean Yiu, Frank Dahlke, Timothy Coetzee, Gary R. Cutter, Steven Yuen, Ulrike Bonati, Fred D. Lublin

**Affiliations:** 1 Research Center for Clinical Neuroimmunology and Neuroscience, University Hospital Basel, University of Basel, Switzerland;; 2 Roche Products Ltd, Welwyn Garden City, United Kingdom;; 3 Impulze GmbH Zürich, Switzerland;; 4 National Multiple Sclerosis Society, New York, NY;; 5 Department of Biostatistics, University of Alabama at Birmingham;; 6 Genentech, Inc., South San Francisco, CA;; 7 F. Hoffmann-La Roche Ltd, Basel, Switzerland; and; 8 Department of Neurology, Icahn School of Medicine at Mount Sinai, New York, NY.

## Abstract

**Background and Objectives:**

Sensitive and meaningful disability worsening measures remain an unmet medical need in multiple sclerosis (MS). Composite confirmed disability worsening/progression (cCDW/cCDP) combines the Expanded Disability Status Scale (EDSS) with performance tests of ambulation and dexterity (Timed 25-Foot Walk Test [T25FWT] and Nine-Hole Peg Test [9HPT]). We assessed the relation of changes in these measures to understand the utility of cCDW/cCDP as an endpoint for MS trials.

**Methods:**

Clinical trials measuring all components of cCDW were selected for the analysis of (i) individual patient-level data from Roche-sponsored MS studies to characterize the association between performance-test changes and subsequent EDSS changes and (ii) population-level data from published studies reporting treatment effects on EDSS and either cCDP or T25FWT or 9HPT events to examine the relationship between treatment effects on T25FWT and EDSS events.

**Results:**

Analysis (i): 6 Roche-sponsored Phase III trials comprising 4,979 patients with relapsing-remitting MS (RRMS; n = 1,225), relapsing MS (RMS; n = 1,656), progressive MS (PMS; n = 922), and primary progressive MS (PPMS; n = 1,171), with a data cutoff of November 2022, were included in the individual patient analyses. For all trials, T25FWT events were associated with increased risk of subsequent EDSS events (hazard ratios [HRs], *p* values: 2.11–5.20, 0.07–<0.001); similar associations were found for 9HPT events with HRs for later EDSS events ranging from 1.47 to 2.66 (*p* values from 0.24–<0.001). For patients without EDSS events in the first 96 study weeks, T25FWT or 9HPT events in the first 96 study weeks were associated with increased risk of subsequent EDSS events (HRs, *p* values: T25FWT 1.74–3.26, 0.01–<0.001; 9HPT 1.45–3.08, 0.45–<0.001). Patients with T25FWT or 9HPT events were more likely to experience a ≥8-point change from baseline at the final visit in the 29-item Multiple Sclerosis Impact Scale physical subscale (risk ratios, *p* values: T25FWT 1.45–2.17, 0.004–<0.001; 9HPT 1.26–1.87, 0.15–0.03). Analysis (ii): In the 9 studies included, treatment effects on T25FWT events were predictive of treatment effects on EDSS events (Spearman correlation [95% CI] = 0.82 [0.34–0.96], *p* = 0.005).

**Discussion:**

In this post hoc analysis, worsening on T25FWT or 9HPT was a harbinger of EDSS worsening and treatment effects on T25FWT correlated with those on EDSS. These results establish the predictive validity and clinical relevance of performance-test worsening, thus supporting use of cCDW/cCDP as a primary outcome for progression in MS trials.

**Clinical Trial Identifiers:**

ClinicalTrials.gov Identifiers: NCT01247324 (OPERA I); first submitted November 23, 2010; first patient enrolled: August 31, 2011; available at clinicaltrials.gov/study/NCT01247324. NCT01412333 (OPERA II); first submitted August 8, 2011; first patient enrolled: September 20, 2011; available at clinicaltrials.gov/study/NCT01412333. NCT03085810 (ENSEMBLE); first submitted March 16, 2017; first patient enrolled: March 27, 2017; available at clinicaltrials.gov/study/NCT03085810. NCT01194570 (ORATORIO); first submitted August 28, 2010; first patient enrolled: March 3, 2011; available at clinicaltrials.gov/study/NCT01194570. NCT03523858 (CONSONANCE); first submitted April 16, 2018; first patient enrolled: May 28, 2018; available at clinicaltrials.gov/study/NCT03523858. NCT00087529 (OLYMPUS); first submitted July 9, 2004; first patient enrolled: July 9, 2004; available at clinicaltrials.gov/study/NCT00087529.

## Introduction

Multiple sclerosis (MS) is a chronic disabling disease of the CNS, clinically characterized by relapses and disability worsening.^1^ Compared with low-to-moderately effective disease-modifying therapies (DMTs) or placebo, highly effective DMTs (e.g., anti-CD20 monoclonal antibodies [MAbs]) substantially reduce relapses and relapse-associated worsening but currently only moderately affect progression independent of relapses, as evidenced by Phase III studies.^2-6^ Therefore, disability accumulation remains an unmet medical need.

The Expanded Disability Status Scale (EDSS) is the established measure of disability in MS and is widely accepted by health authorities.^7,8^ However, it has significant limitations, including its ordinal character, low reliability, and low sensitivity to change.^9-11^ Insensitivity to change at certain disability levels leads to varying transition times between EDSS steps (with a typically bimodal distribution),^12,13^ contributing to low event rates of confirmed increases in disability based on the EDSS (henceforth EDSS events). In MS trials using more efficacious comparator DMTs, EDSS event rates decrease further, requiring larger sample sizes and/or longer study durations to demonstrate a treatment effect with reasonable statistical power (eFigure 1). Responding to this challenge for conducting trials, the US Food and Drug Administration (FDA) has developed guidance on multiple endpoints in clinical trials and supports using composite endpoints to “provide a substantially higher overall event rate that allows a study with reasonable sample size and study duration to have adequate power” in indications where event rates are low.^14^ This approach has already been critically reviewed and partially adopted by some health authorities in Alzheimer disease.^15,16^

The Multiple Sclerosis Functional Composite (MSFC) was proposed as an endpoint in MS to compensate for weaknesses of the EDSS by including quantitated performance tests of clinically relevant domains not or only partly addressed by the EDSS.^17^ The MSFC combines 2 widely used performance tests (Nine-Hole Peg Test [9HPT] and Timed 25-Foot Walk Test [T25FWT]) to assess upper extremity dexterity and walking speed, with a test of cognitive function (initially the Paced Auditory Serial Addition Test, subsequently replaced by the Symbol Digit Modalities Test [SDMT]).^18,19^ However, up to now, the MSFC has not been broadly accepted as a primary endpoint in Phase III MS trials, mainly because of issues with clinical interpretability and meaningfulness of the overall z-score.^11,20^ For instance, while the thresholds for clinically relevant change in the EDSS are established, the proposed clinically relevant^21,22^ change in the performance tests (confirmed ≥20% worsening of T25FWT and 9HPT scores, henceforth T25FWT and 9HPT events) was less broadly accepted. In a recent qualification of a clinical composite endpoint including T25FWT and 9HPT by the Multiple Sclerosis Outcome Assessments Consortium (MSOAC), this threshold was again proposed and interpreted as meaningful.^23,24^ Based on these considerations, composite confirmed disability worsening (cCDW), which combines EDSS and performance tests, was proposed. This composite endpoint has also been used as a primary endpoint under the name “composite confirmed disability progression (cCDP)” in 2 Phase III trials (fingolimod in primary progressive multiple sclerosis [PPMS]^25^; natalizumab in secondary progressive multiple sclerosis [SPMS])^4^ and, more recently, in ongoing trials of higher dose ocrelizumab (OCR) in relapsing multiple sclerosis (RMS) and PPMS (NCT04544436, NCT04548999), fenebrutinib in PPMS (NCT04544449), tolebrutinib in PPMS (NCT04458051), and frexalimab in nonrelapsing SPMS (NCT06141486) and the second stage of the multiarm, multistage OCTOPUS trial (ISRCTN14048364). Very recently, again using the large MSOAC placebo database, it was shown that patients with T25FWT events were more likely to experience EDSS events, and that T25FWT events occurred before EDSS events 65% of the time.^26^

To further assess the predictive value and meaningfulness of cCDW/cCDP, we quantify the risk of EDSS events after the onset of performance-test events using individual patient data from 6 large clinical trials of DMTs, comprising 4,979 patients across MS clinical courses. We also examine the relationship between treatment effects on T25FWT and EDSS events in a combined analysis of published data from 9 clinical trials including a wide spectrum of MS clinical courses and grades of disability. Finally, we investigate patient-reported outcomes (PROs) for patients with and without performance-test events to further validate their patient relevance.

## Methods

### Trial Design and Procedures

Clinical trials that measured all components of CDW were selected for analysis of (i) individual patient-level data from all Roche-sponsored MS studies (data on file) and (ii) aggregate data, identified using a meta-analysis of randomized trials method.^27^ In particular, PubMed was used to identify all MS clinical trials reporting treatment effects on EDSS and either cCDP or T25FWT or 9HPT events, and data were collected from the corresponding study publications.

### Standard Protocol Approvals, Registrations, and Patient Consent

The trial protocols (ClinicalTrials.gov identifier numbers: OPERA I, NCT01247324; OPERA II, NCT01412333; ENSEMBLE, NCT03085810; ORATORIO, NCT01194570; CONSONANCE, NCT03523858; and OLYMPUS, NCT00087529) were approved by the relevant institutional review boards (IRBs)/ethics committees (IECs). All patients provided written informed consent on dedicated forms (ICFs). For Roche studies, this analysis was exempt from IRB/IEC review because it was already covered by the IRB/IEC approvals and ICFs of the individual studies.

### Study Disability Assessments and Definitions

cCDW, also often known under the name cCDP in clinical trials, was defined identically, that is, either an EDSS^28^ increase of ≥1.0 points if the baseline EDSS score was ≤5.5 points or ≥0.5-point increase if the baseline EDSS score was >5.5 points, or an increase of ≥20% in T25FWT or 9HPT scores, respectively, confirmed after 12 or 24 weeks (9HPT times were analyzed according to the MSFC manual.^29^ Specifically, the 2 trials for each hand were averaged and converted to the reciprocals of the mean times for each hand and then the 2 reciprocals were averaged, to which the 20% worsening was applied). A cCDW event refers to the first occurrence of cCDW. The physical impact of MS on day-to-day life was assessed using the 29-item Multiple Sclerosis Impact Scale (MSIS-29) physical subscale.^30^

### Statistical Analyses

The time of cCDW and individual component events indicated the first time where cCDW and the individual component events occurred. For patients with PPMS and progressive multiple sclerosis (PMS), an initial event was imputed as a confirmed event if missing follow-up data (e.g., due to dropout from study) did not permit confirmation of the initial event. Statistical hypotheses were tested at the 5% significance level (α = 0.05) against 2-sided alternatives. The following analyses were performed.

#### Analysis 1

Characterizing the frequency and timing of events: The number and proportion of patients with cCDW and each of the individual component events and the number and proportion of times that individual component events were leading events, that is, occur as the first event in the composite, were analyzed.

#### Analysis 2

Quantifying the change in risk of EDSS events after the onset of performance-test events: The change in risk of EDSS events, as characterized by the hazard ratio (HR), after the onset of T25FWT and 9HPT events was estimated separately in each study arm to determine potential harbinger effects of performance-test events on EDSS events. This analysis was performed by fitting a time-varying exposure Cox model with presence/absence of T25FWT and 9HPT events as time-varying covariates and EDSS score, age (years), sex, and disease duration (years) as baseline covariates.

The following sensitivity analyses were also performed to understand the robustness of the results: The confirmation period for EDSS events was extended to 48 or more weeks. For the association between previous T25FWT and EDSS events, the EDSS score was derived by excluding the ambulation score, thus restricting the EDSS score to 0–5 (eTable 1 and eFigure 2). This analysis provides insights into whether potential harbinger effects of T25FWT events on EDSS events are not only driven by changes in the ambulation score but also applicable to changes in other functional systems scores. The alternative approach of focusing on patients with baseline EDSS score ≤4 (i.e., with limited restriction in ambulation) was not considered because the inclusion criteria for the PMS studies (baseline EDSS score ≥3.5) would result in a very low number of patients contributing to the analysis.

To further establish the predictive validity of performance-test events within a 96-week time frame, the differences in risk of EDSS events confirmed for 24 or more weeks in patients with vs without T25FWT and 9HPT events during the first 96 weeks of the study were estimated for the subset of patients with long-term EDSS events (i.e., occurring after the first 96 weeks of the study). This analysis was performed by fitting a Cox model with presence/absence of early T25FWT and 9HPT events as time-independent covariates and EDSS score, age (years), sex, and disease duration from diagnosis (years) as baseline covariates.

#### Analysis 3

Examining the relationship between treatment effects on T25FWT and EDSS events: Using the studies identified in analysis (ii), the relationship between the treatment effects on T25FWT and EDSS events, and cCDW and EDSS events were calculated using the Spearman rank correlation coefficient.

In addition, cross validation was performed to assess whether treatment effects on T25FWT events are predictive of treatment effects on EDSS events. Specifically, the following steps were applied to each study: (1) the respective study was set aside as a test study, (2) a linear model was fitted to the remaining studies with a log HR for EDSS events as the outcome and a log HR for T25FWT events and study population (RMS or RMS vs SPMS or PPMS) as covariates, and (3) the fitted linear model and the HR for T25FWT events from the test study were used to predict the HR for EDSS events for the test study.

#### Analysis 4

Characterizing PROs by performance-test event status: For each scheduled visit (n = 4) over 144 weeks with sufficient data (at least 75% of patients), the number and proportion of patients with ≥8-point increase (minimal clinically important difference score)^31,32^ from baseline in the MSIS-29 physical subscale were estimated for patients with and without T25FWT and 9HPT events by that visit. The risk ratio, that is, the chance of experiencing a ≥8-point increase in the MSIS-29 physical subscale for patients with T25FWT/9HPT events divided by the chance of experiencing such an increase for patients without T25FWT/9HPT events, was calculated. This analysis was performed by fitting generalized estimating equation models for binary outcomes with unstructured correlation matrices to capture within-patient correlation, presence of a ≥8-point increase from baseline in MSIS-29 physical subscale as the outcome, and presence of T25FWT and 9HPT events as the independent variable.

### Data Availability

Qualified researchers may request access to individual patient-level data through the clinical study data request platform (vivli.org/ourmember/roche/).^33^ Further details on Roche's Global Policy on the Sharing of Clinical Information and how to request access to related clinical study documents can be found at go.roche.com/data_sharing.^34^ Anonymized records for individual patients across more than one data source external to Roche cannot, and should not, be linked because of a potential increase in risk of patient re-identification.

## Results

### Studies Included in the Analysis

Six Roche-sponsored Phase III trials, comprising 4,979 patients with relapsing-remitting multiple sclerosis (RRMS), RMS, PMS, and PPMS, with a data cutoff of November 2022, were included in the analyses (completed: OPERA I/II plus open-label extension [OLE] [RMS, n = 1,656, OCR vs interferon (IFN) β-1a],^2,35^ ORATORIO plus OLE [PPMS, n = 732, OCR vs placebo],^3,36^ OLYMPUS [PPMS, n = 439, rituximab vs placebo]^37^; ongoing: ENSEMBLE [RRMS, n = 1,225, OCR single-arm study],^38^ CONSONANCE [PMS, n = 922, OCR single-arm study]^39^; eTable 2). Key inclusion criteria included an age of 18–55 years (OPERA I/II, ORATORIO, ENSEMBLE) or 18–65 years (CONSONANCE, OLYMPUS) and an EDSS score of 0–5.5 (OPERA I/II), 3–6.5 (ORATORIO), 0–3.5 (ENSEMBLE), <6.5 (CONSONANCE), or 2–6.5 points (OLYMPUS) at screening. ENSEMBLE patients with RRMS also must have had <3 years of disease duration from first clinical attack. Baseline demographics and disease characteristics were typical of the MS populations selected for the respective studies and are provided in Table 1.

**Table 1:** Baseline Characteristics by Study Arms

Baseline characteristics^a^	ENSEMBLE (N = 1,225)	OPERA I/II OCR (N = 827)	OPERA I/II IFN (N = 829)	CONSONANCE (N = 629)	ORATORIO OCR (N = 488)	ORATORIO PBO (N = 244)	OLYMPUS RTX (N = 292)	OLYMPUS PBO (N = 147)
Age, y	32.8 ± 9.1	37.1 ± 9.2	37.2 ± 9.2	48.5 ± 9.2	44.7 ± 7.9	44.4 ± 8.3	50.1 ± 9.0	49.6 ± 8.7
Female, n (%)	784 (64.0)	541 (65.4)	552 (66.6)	329 (52.3)	237 (48.6)	124 (50.8)	140 (47.9)	81 (55.1)
MS phenotype, n (%)								
RMS^b^	1,225 (100.0)	827 (100.0)	829 (100.0)	—	—	—	—	—
PPMS	—	—	—	305 (48.5)	488 (100.0)	244 (100.0)	292 (100.0)	147 (100.0)
SPMS^b^	—	—	—	324 (51.5)	—	—	—	—
Geographic region, n (%)								
EU/Switzerland/Norway	735 (60.0)	401 (48.5)	385 (46.4)	388 (61.7)	315 (64.5)	157 (64.3)	0 (0.0)	0 (0.0)
Latin America	107 (8.7)	45 (5.4)	61 (7.4)	64 (10.2)	16 (3.3)	6 (2.5)	0 (0.0)	0 (0.0)
Non-EU/Middle East/Africa	58 (4.7)	123 (14.9)	117 (14.1)	68 (10.8)	61 (12.5)	32 (13.1)	0 (0.0)	0 (0.0)
United States/Canada/Australia/New Zealand	325 (26.5)	258 (31.2)	266 (32.1)	109 (17.3)	96 (19.7)	49 (20.1)	292 (100.0)	147 (100.0)
Time since symptom onset, y	1.1 ± 0.9	6.7 ± 6.2	6.5 ± 6.1	11.7 ± 8.0	6.7 ± 4.0	6.1 ± 3.6	9.5 ± 6.5	9.4 ± 6.9
Time since diagnosis, y	0.3 ± 0.4	4.0 ± 4.9	3.9 ± 4.9	7.9 ± 7.2	2.9 ± 3.2	2.9 ± 3.2	4.2 ± 4.3	3.9 ± 4.3
Number of relapses in previous 12 mo	1.4 ± 0.8	1.3 ± 0.7	1.3 ± 0.7	0.1 ± 0.4	0.0 ± 0.0	0.0 ± 0.0	0.0 ± 0.0	0.0 ± 0.0
Previous DMT, n (%)	10 (0.8)	222 (26.8)	219 (26.4)	380 (60.4)	55 (11.3)	29 (11.9)	28 (9.6)	10 (6.8)
EDSS, score	1.8 ± 0.9	2.8 ± 1.3	2.8 ± 1.3	5.0 ± 1.3	4.7 ± 1.2	4.7 ± 1.2	4.8 ± 1.3	4.7 ± 1.4
T25FWT, s	6.3 ± 5.9	7.9 ± 9.9	7.2 ± 9.2	15.1 ± 18.1	14.8 ± 21.2	12.9 ± 15.5	13.5 ± 21.1	12.5 ± 18.9
9HPT, s	22.0 ± 7.5	24.5 ± 13.1	24.0 ± 8.3	32.6 ± 15.7	31.9 ± 23.3	30.6 ± 13.4	31.4 ± 27.3	28.9 ± 11.2
Presence of T1 Gd-enhancing lesions, no/total n (%)	568/1,220 (46.6)	333/818 (40.7)	327/822 (39.8)	103/628 (16.4)	133/484 (27.5)	60/243 (24.7)	70/290 (24.1)	37/147 (25.2)
Normalized brain volume,^c^ cm^3^	1,460.4 ± 81.3	1,502.4 ± 88.5	1,500.2 ± 89.3	1,498.3 ± 84.7	1,462.9 ± 84.0	1,469.9 ± 88.7	1,203.9 ± 120.5	1,209.5 ± 127.4

Abbreviations: 9HPT = Nine-Hole Peg Test; aSPMS = active secondary progressive multiple sclerosis; CIS = clinically isolated syndrome; DMT = disease-modifying therapy; EDSS = Expanded Disability Status Scale; EU = European Union; Gd = gadolinium; IFN = interferon β-1a; MS = multiple sclerosis; OCR = ocrelizumab; PBO = placebo; PPMS = primary progressive multiple sclerosis; RMS = relapsing multiple sclerosis; RTX = rituximab; SPMS = secondary progressive multiple sclerosis; T25FWT = Timed 25-Foot Walk Test.

a Plus–minus values are means ± SD.

b RMS includes CIS, RRMS, and aSPMS.

c Baseline assessments were performed at 8 wk after treatment with OCR in ENSEMBLE.

After review of the corresponding study publications from studies identified on PubMed,^27^ the following studies were selected: AFFIRM (NCT00027300 [RRMS, n = 942, natalizumab vs placebo]),^40^ ASCEND (NCT01416181 [SPMS, n = 889, natalizumab vs placebo]),^41^ EXPAND (NCT01665144 [SPMS, n = 1,652, siponimod vs placebo]),^42^ INFORMS (NCT00731692 [PPMS, n = 970, fingolimod vs placebo]),^25^ OLYMPUS (PPMS),^29^ OPERA I/II (RMS),^2^ ORATORIO (PPMS),^3^ and PROMISE (PPMS, n = 943, glatiramer acetate vs placebo)^43^ (eTable 3). Please note that of the studies identified, EXPAND and PROMISE did not report treatment effects on cCDW.

### Timing of Assessments Across Roche-Sponsored Phase III Trials

EDSS^28^ scores were assessed every 12 weeks during the double-blind treatment period (DBT) phase of OPERA I/II, ORATORIO, and OLYMPUS, and the OLE of ORATORIO and every 24 weeks during the OLE of OPERA I/II, ENSEMBLE, and CONSONANCE. T25FWT and 9HPT followed the same schedule as EDSS in all studies but were not performed in the OLE of OPERA I/II. Assessments of the MSIS-29 physical subscale were not performed in OPERA I/II, ORATORIO, or OLYMPUS, and occurred every 48 weeks in ENSEMBLE and CONSONANCE. Unless specified otherwise, a 12-week confirmation period was used for cCDW events for OPERA I/II DBT, ORATORIO DBT, and OLYMPUS, in line with the original study protocols. A 24-week confirmation period was used for OPERA I/II DBT and OLE, ORATORIO DBT and OLE, ENSEMBLE, and CONSONANCE. Although assessments were scheduled to occur every 12/24 weeks, it was possible for patients to have unscheduled assessments at any time. A continuous time approach is required to account for the unscheduled assessments. For this reason, continuous time approaches are typically used to estimate treatment effects in clinical trials.

### Characterizing the Frequency and Timing of Events (Analysis 1)

Across the RMS and PMS studies, cCDW/cCDP events occurred 1.66 (309/186) to 2.21 (166/75) times more frequently than EDSS events (Table 2). A greater proportion of patients had T25FWT events (12.3%–61.5%) compared with EDSS events (9.1%–43.9%) and 9HPT events (3.0%–29.9%). The proportion of patients with events was lower in RMS studies compared with PMS studies for cCDW (20.1%–28.1% vs 54.2%–73.1%), T25FWT (12.3%–17.9% vs 39.3%–61.5%), EDSS (9.1%–13.9% vs 27.7%–43.9%), and 9HPT (3.0%–4.0% vs 12.7%–29.9%). Similarly, the proportion of patients with events in multiple (2 or 3) components of cCDW was infrequent and lower in RMS compared with PMS studies (2 components: RMS 3.6%–5.2%, PMS 17.8%–25.2%; 3 components: RMS 0.4%–1.2%, PMS 5.1%–18.4%; Table 2). In PMS studies, where imputation was applied, the number of cCDW events and their individual components was largely similar with or without imputation (eTable 4). The relative frequency of cCDW vs EDSS events did not change when 24-week confirmed events were considered (eTable 5).

**Table 2:** Number and Proportion of Patients With cCDW and Individual Component Events

Study	Number (%) of patients with cCDW and individual component events per study arm				Number (%) of patients with events in multiple components per study arm		Relative frequency of cCDW vs EDSS events
	T25FWT	EDSS	9HPT	cCDW	Two components	All 3 components	
RMS studies							
OPERA I/II OCR DBT (N = 827)	102 (12.3)	75 (9.1)	25 (3.0)	166 (20.1)	30 (3.6)	3 (0.4)	2.21 (166/75)
OPERA I/II IFN DBT (N = 829)	127 (15.3)	112 (13.5)	31 (3.7)	222 (26.8)	40 (4.8)	4 (0.5)	1.98 (222/112)
ENSEMBLE^a^ (N = 1,225)	219 (17.9)	170 (13.9)	49 (4.0)	344 (28.1)	64 (5.2)	15 (1.2)	2.02 (344/170)
PMS studies							
ORATORIO OCR DBT (N = 488)	257 (52.7)	186 (38.1)	102 (20.9)	309 (63.3)	112 (23.0)	62 (12.7)	1.66 (309/186)
ORATORIO PBO DBT (N = 244)	150 (61.5)	107 (43.9)	73 (29.9)	180 (73.8)	60 (24.6)	45 (18.4)	1.68 (180/107)
OLYMPUS RTX DBT (N = 292)	121 (41.4)	83 (28.4)	37 (12.7)	159 (54.5)	52 (17.8)	15 (5.1)	1.92 (159/83)
OLYMPUS PBO DBT (N = 147)	77 (52.4)	58 (39.5)	27 (18.4)	97 (66.0)	37 (25.2)	14 (9.5)	1.67 (97/58)
CONSONANCE^a^ (N = 629)	247 (39.3)	174 (27.7)	109 (17.3)	341 (54.2)	129 (20.5)	30 (4.8)	1.96 (341/174)

Abbreviations: 9HPT = Nine-Hole Peg Test; cCDW = composite confirmed disability worsening; DBT = double-blind treatment period; EDSS = Expanded Disability Status Scale; IFN = interferon β-1a; OCR = ocrelizumab; PBO = placebo; PMS = progressive multiple sclerosis; RMS = relapsing multiple sclerosis; RTX = rituximab; T25FWT = Timed 25-Foot Walk Test.

Note that patients can contribute to several columns.

a The confirmation period for events was at least 6 (as opposed to 3) months for these studies.

In all study arms, more patients experienced T25FWT events before or without EDSS events (55.8%–67.9%) compared with EDSS events before or without T25FWT events (22.7%–39.5%), particularly in PMS studies (Table 3). Of the patients with baseline EDSS score ≥4 (the range where changes become heavily weighted toward the ambulation score), 9HPT events occurred earlier than EDSS events in 38.7%–46.7% and 22.4%–39.2% in RMS and PMS studies, respectively (Table 3). Despite T25FWT events often occurring earlier and more frequently than EDSS events, EDSS events remain a relevant contributor to cCDW, constituting 38.1%–39.7% and 26.7%–31.1% of cCDW events in RMS and PMS studies, respectively (Table 3).

**Table 3:** Sequence of the Individual Component Events of cCDW

Study	Timing of T25FWT events relative to EDSS^a^ events in each study arm (%)			No. of patients with 9HPT events before or without EDSS events in patients with baseline EDSS score ≥4^b^ in each study arm (%)	Individual component events as the leading event^c^ in each study arm (%)		
	T25FWT events before or without EDSS events	EDSS events before or without T25FWT events	EDSS events at the same time as T25FWT events		T25FWT events	EDSS events	9HPT events
RMS studies							
OPERA I/II OCR DBT	87/151 (57.6)	60/151 (39.7)	4/151 (2.6)	7/15 (46.7)	90/176 (51.1)	67/176 (38.1)	19/176 (10.8)
OPERA I/II IFN DBT	112/195 (57.4)	77/195 (39.5)	6/195 (3.1)	12/31 (38.7)	117/229 (51.1)	91/229 (39.7)	21/229 (9.2)
ENSEMBLE^d^	178/319 (55.8)	126/319 (39.5)	15/319 (4.7)	NA	192/365 (52.6)	142/365 (38.9)	31/365 (8.5)
PMS studies							
ORATORIO OCR DBT	188/277 (67.9)	63/277 (22.7)	26/277 (9.4)	50/157 (31.8)	213/341 (62.5)	91/341 (26.7)	37/341 (10.9)
ORATORIO PBO DBT	99/160 (61.9)	47/160 (29.4)	14/160 (8.8)	29/88 (33.0)	116/203 (57.1)	59/203 (29.1)	28/203 (13.8)
OLYMPUS RTX DBT	94/140 (67.1)	41/140 (29.3)	5/140 (3.6)	13/58 (22.4)	102/167 (61.1)	52/167 (31.1)	13/167 (7.8)
OLYMPUS PBO DBT	52/82 (63.4)	22/82 (26.8)	8/82 (9.8)	17/52 (32.7)	62/111 (55.9)	32/111 (28.8)	17/111 (15.3)
CONSONANCE^d^	203/321 (63.2)	102/321 (31.8)	16/321 (5.0)	74/189 (39.2)	198/371 (53.4)	106/371 (28.6)	67/371 (18.1)

Abbreviations: 9HPT = Nine-Hole Peg Test; cCDW = composite confirmed disability worsening; DBT = double-blind treatment period; EDSS = Expanded Disability Status Scale; IFN = interferon β-1a; NA = not applicable; OCR = ocrelizumab; PBO = placebo; PMS = progressive multiple sclerosis; RMS = relapsing multiple sclerosis; RTX = rituximab; T25FWT = Timed 25-Foot Walk Test.

The denominator for calculating proportions is as follows:

a The number of patients with T25FWT and/or EDSS events.

b The number of patients with baseline EDSS score ≥4, and EDSS and/or 9HPT events.

c The number of patients with cCDW.

d The confirmation period for EDSS, T25FWT, or 9HPT events was at least 6 (as opposed to 3) months for these studies.

### Performance-Test Events and Risk of Subsequent EDSS Events (Analysis 2)

In all study arms, T25FWT events were strongly and positively associated with an increased risk of EDSS events, particularly in PMS studies, where baseline EDSS values were higher (HR, [*p* value] ranges: RMS, 2.11–5.20 [0.07–<0.001]; PMS, 2.95–5.20 [all ≤0.001] Table 4). Apart from the ENSEMBLE study and the OCR arm of the pooled OPERA I/II studies where 9HPT events were rare, 9HPT events were also associated with an increased risk of EDSS events, again, particularly in PMS studies (HR, [*p* value] ranges: PMS, 1.47–2.66 [0.24–<0.001], statistical significance in 4 of 7 study arms). The results were broadly similar when a 48-week confirmation period was used for EDSS events (details in eTable 6). To limit the contribution of gait disturbances to EDSS events, the associations between T25FWT and EDSS events were reassessed after excluding the ambulation score from the EDSS calculation. For all but one RMS and PMS study arm, respectively, the strong positive associations remained largely unchanged (eTable 7).

**Table 4:** Hazard Ratios (HRs, 95% CI, *p* value) for EDSS Events After T25FWT or 9HPT Events

Study	Risk of EDSS events with vs without previous T25FWT events (HR, 95% CI, *p* value)	Risk of EDSS events with vs without previous 9HPT events (HR, 95% CI, *p* value)
RMS studies		
OPERA I/II OCR DBT	2.11 (0.94–4.71); *p* = 0.07	0.62 (0.07–5.21); *p* = 0.66
OPERA I/II IFN DBT	2.56 (1.53–2.29); *p* < 0.001	2.48 (1.00–6.19); *p* = 0.05
ENSEMBLE^a^	2.29 (1.50–3.49); *p* < 0.001	1.23 (0.30–5.02); *p* = 0.77
PMS studies		
ORATORIO OCR DBT	5.20 (3.63–7.45); *p* < 0.001	2.66 (1.75–4.05); *p* < 0.001
ORATORIO OCR DBT + OLE^b^	4.33 (3.44–5.77); *p* < 0.001	2.41 (1.77–2.39); *p* < 0.001
ORATORIO PBO DBT	3.44 (2.13–5.57); *p* < 0.001	1.47 (0.78–2.79); *p* = 0.24
ORATORIO PBO DBT + OLE^b^	4.03 (2.70–6.03); *p* < 0.001	1.53 (0.97–2.40); *p* = 0.07
OLYMPUS RTX DBT	3.67 (2.13–6.35); *p* < 0.001	2.48 (0.96–6.41); *p* = 0.06
OLYMPUS PBO DBT	3.29 (1.73–6.25); *p* = 0.001	2.36 (1.08–5.13); *p* = 0.03
CONSONANCE^a^	2.99 (2.05–4.37); *p* < 0.001	1.87 (1.18–2.95); *p* = 0.008

Abbreviations: 9HPT = Nine-Hole Peg Test; DBT = double-blind treatment period; EDSS = Expanded Disability Status Scale; HR = hazard ratio; IFN = interferon β-1a; OCR = ocrelizumab; OLE = open-label extension; PBO = placebo; PMS = progressive multiple sclerosis; RMS = relapsing multiple sclerosis; RTX = rituximab; T25FWT = Timed 25-Foot Walk Test.

a The confirmation periods for T25FWT, EDSS, and 9HPT events were at least 24 wk (as opposed to 12 wk) for these studies.

b The confirmation period for EDSS events was at least 24 wk, and for T25FWT and 9HPT events, the confirmation periods were at least 12 wk.

**Figure 1:**
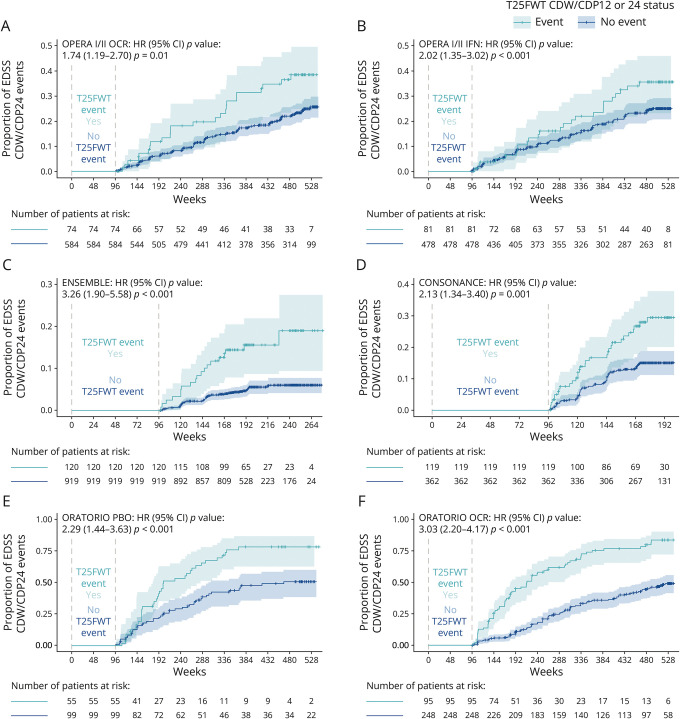
Kaplan-Meier Estimates of the Proportion of Patients With EDSS Events Confirmed for At Least 24 Weeks Regarding Patients With and Without T25FWT Events Confirmed for At Least 12 Weeks (OPERA [A, B] and ORATORIO [E, F]) or 24 Weeks (ENSEMBLE [C] and CONSONANCE [D]) in the First 96 Weeks of the Studies CDP = confirmed disability progression; CDW = confirmed disability worsening; EDSS = Expanded Disability Status Scale; HR = hazard ratio; IFN = interferon β-1a; OCR = ocrelizumab; PBO = placebo; T25FWT = Timed 25-Foot Walk Test.

In the subsets of patients without EDSS events in the first 96 weeks, strong positive and statistically significant associations between occurrence of T25FWT events in the first 96 weeks of the study and subsequent EDSS events were seen (risk of future EDSS events, HR [95% confidence interval (CI); *p* value]: OPERA I/II OCR, 1.74 [1.12–2.70; *p* = 0.01]; OPERA I/II IFN β-1a, 2.02 [1.35–3.02; *p* < 0.001]; ENSEMBLE, 3.26 [1.90–5.58; *p* < 0.001]; ORATORIO OCR, 3.03 [2.20–4.17; *p* < 0.001]; ORATORIO placebo [PBO], 2.29 [1.44–3.63; *p* < 0.001]; CONSONANCE, 2.13 [1.34–3.40; *p* = 0.001]; Figure 1). Similarly, the occurrence of 9HPT events in the first 96 study weeks was associated with an increased risk of subsequent EDSS events in patients of all eligible study arms (apart from the OCR arm of OPERA I/II), and the associations were stronger in PMS studies (risk of future EDSS events; HR [95% CI; *p* value]: OPERA I/II IFN, 1.45 [0.66–3.16; *p* = 0.35]; ENSEMBLE, 2.14 [0.30–15.47; *p* = 0.45]; ORATORIO OCR, 3.08 [1.99–4.78; *p* < 0.001]; ORATORIO PBO, 2.03 [1.12–3.69; *p* = 0.02]; CONSONANCE, 2.10 [1.13–3.91; *p* = 0.02]).

### Relationship Between Treatment Effects on T25FWT and EDSS Events (Analysis 3)

**Figure 2:**
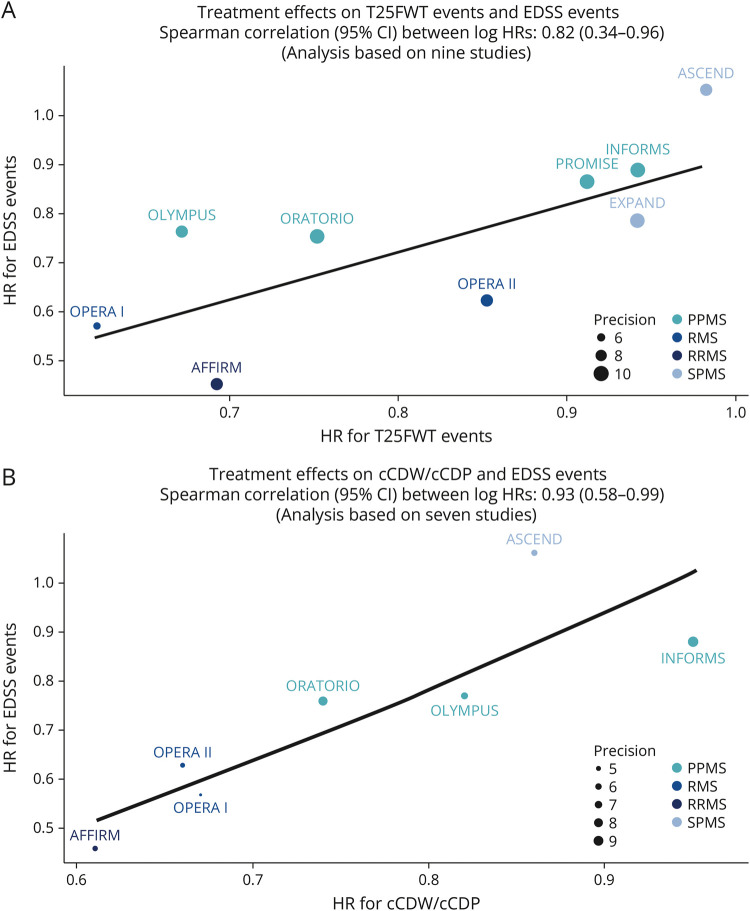
Plot of Treatment Effects on (A) T25FWT and EDSS Events and (B) cCDW/cCDP and EDSS Events on the Log-Hazard Ratio Scale From Nine/Seven Studies Across Multiple Sclerosis Phenotypes 9HPT = Nine-Hole Peg Test; cCDP = composite confirmed disability progression; cCDW = composite confirmed disability worsening; CDW12 = confirmed disability worsening at 12 weeks; CDW24 = confirmed disability worsening at 24 weeks; EDSS = Expanded Disability Status Scale; HR = hazard ratio; PPMS = primary progressive multiple sclerosis; RMS = relapsing multiple sclerosis; RRMS = relapsing-remitting multiple sclerosis; SPMS = secondary progressive multiple sclerosis; T25FWT = Timed 25-Foot Walk Test. Note that treatment effects for AFFIRM are based on CDW24, and treatment effects on T25FWT-CDW for PROMISE are based on a composite between T25FWT-CDW and 9HPT-CDW. Otherwise, all other treatment effects are for T25FWT-CDW12 and EDSS-CDW12.

The analysis of 9 published studies including patients with RMS or PMS showed that treatment effects on T25FWT and cCDW were highly correlated with treatment effects on EDSS (Spearman rank correlation coefficient [95% CI]: 0.82 [0.34–0.96] *p* = 0.005, and 0.93 [0.58–0.99] *p* < 0.001, respectively; Figure 2; note that the correlation between the treatment effects on cCDW and EDSS events was based on 7 of the 9 studies because treatment effects on cCDW were not reported in EXPAND and PROMISE). The results of the cross-validation analysis showed that all predicted treatment effects on EDSS events were within the reported 95% CIs (eFigure 3). The correlation between the treatment effects on EDSS and 9HPT events based on 7 studies was low (Spearman correlation = −0.037; 95% CI = [−0.77 to 0.74]), which was not surprising given the low incidence of 9HPT events and the known limitations of the EDSS in capturing dexterity.

### Comparison of MSIS-29 Physical Subscale Outcomes for Patients With and Without T25FWT or 9HPT Events (Analysis 4)

At each visit (n = 4) in ENSEMBLE and CONSONANCE, a higher proportion of patients had ≥8-point change from baseline in the MSIS-29 physical subscale for patients with vs without T25FWT events and with vs without 9HPT events (risk ratio [*p* value] ranges: ENSEMBLE, T25FWT 2.17–4.06 [all <0.001] and 9HPT 1.87–3.52 [0.08–0.001]; CONSONANCE, T25FWT 1.26–1.71 [0.30–0.001] and 9HPT 1.21–1.79 [0.33–0.03]; eTable 8).

## Discussion

Composite endpoints in clinical trials are being considered in diseases that may affect different functions separately and in settings where incidences of events for individual endpoints may be too low to have adequate statistical power without significantly inflating sample sizes.^14^ These analyses demonstrating the predictive validity of performance-test events for subsequent EDSS events strongly support their additional value and utility and justify the use of cCDW/cCDP as a sensitive and comprehensive endpoint of disability worsening in clinical trials.

In recent Phase III drug development programs in RMS, pooling of 2 identical studies was applied to compensate for the lower statistical power due to the low sensitivity of the EDSS and lower number of EDSS events under treatment with active comparators of proven efficacy.^2,5^ Using high-efficacy drugs, such as anti-CD20 MAbs, in the comparator arm of Phase III studies, would further accentuate this shortcoming and necessitate sample sizes and study durations placing significant burden on patients and investigators, thereby challenging study feasibility (eFigure 1). Introduction of the more sensitive but still well-defined and meaningful cCDW as an endpoint will improve the sensitivity for disability events and thus reduce required sample sizes. The performance tests, T25FWT and 9HPT, increase sensitivity and complement the EDSS by assessing clinically relevant domains of disease worsening that only partially overlap with the EDSS but are still highly predictive of later confirmed EDSS increases. Their utility as part of a composite measure is further supported by a number of studies that have proven their reliability and clinical relevance for patients with MS across various disability levels.^21-23,44-46^

In line with previous studies^16^ and across all MS clinical courses and treatment arms investigated, the T25FWT was the strongest contributor to cCDW, constituting approximately 50%–60% of all events across all study arms. However, EDSS events also contributed substantially to the composite, ranging 38.1%–39.7% and 26.7%–31.1% in the RMS and PMS studies, respectively (Table 1), indicating that cCDW is not wholly driven by changes in the performance tests. While dexterity, as measured by the 9HPT, did not add significantly to first progression events in the R(R)MS study populations, it did so in the PMS studies and, importantly, across all MS clinical courses in patients with EDSS score ≥4. In this analysis, EDSS events are driven almost entirely by ambulation, although changes in the upper limbs are not considered, despite the critical importance of hand and arm functions for patients. The 9HPT is, therefore, a valuable addition to the composite, particularly in patients with PMS and all patients with higher EDSS scores. In future studies, alternative definitions of 9HPT events that result in more events^47^ could be explored. In the CONSONANCE study, which included both nonactive patients with SPMS and PPMS and required 6-month confirmation, the number of events per patient and the event frequency were between the R(R)MS and PPMS studies.

By contrast to a previous study investigating the predictive value of T25FWT events for subsequent EDSS events,^25^ in this study, data from active treatment arms across MS clinical courses, longer term follow-up beyond 96 weeks, and temporal ordering of events were used to demonstrate the harbinger relationship of performance-test events with EDSS events. Specifically, in all study arms and particularly PMS studies, the onset of T25FWT and 9HPT events was strongly and positively associated with an increased risk of confirmed EDSS events, which, only when these events were rare, did not reach statistical significance. The broadly similar findings of sensitivity analysis with the stricter 48-week confirmation period for EDSS events strengthen the findings (eTable 6).

Of interest, the strong positive association of previous T25FWT events with subsequent EDSS events generally remained unchanged after exclusion of the ambulation score in the calculation of Neurostatus EDSS^28^ events and subsequently defined EDSS events (eTable 7). These results suggest that T25FWT events might be associated with a generally increased risk of worsening in functional systems outside ambulation. Prevention of worsening in the performance tests using highly effective DMTs may, therefore, also prevent subsequent nonambulation-related EDSS events, underlining the relevance of T25FWT as a harbinger of subsequent EDSS events, as consistently shown in all 6 studies with longer follow-up. Similarly, 9HPT events in the first 96 weeks were harbingers of later first-time EDSS events, if they occurred at a higher frequency such as in the patients with PMS.

The close log-linear relationship and high Spearman rank correlation coefficients of 0.82 and 0.93 between treatment effects on T25FWT or cCDW and EDSS events (Figure 2) in 7 and 9 randomized studies, respectively, from the literature review demonstrated a strong consistency of treatment effects on the outcomes. Reasonably accurate predictions of EDSS treatment effects using T25FWT treatment effects further support their strong association. Overall, these results suggest that treatment effects on T25FWT, cCDW, and EDSS events may be independently capturing the effect of the treatment on aspects of the same underlying pathophysiologic processes driving disability progression.

The findings of these analyses support the notion that worsening on the performance tests affects daily living for patients with MS. Specifically, interrogation of the ENSEMBLE (early RMS) and CONSONANCE (PMS) data sets revealed that patients who experienced a T25FWT event had a higher risk of reporting clinically meaningful change on the MSIS-29 physical subscore (≥8-point change from baseline),^31,32^ particularly patients with early RMS. Directionally similar results were obtained for 9HPT.

A major strength of this study is the very large data set from high-quality Good Clinical Practice–compliant Phase III and Phase IIIb studies. Studies across different MS clinical courses and treatments allowed assessment of the consistency of findings across a broad range of settings relevant for clinical trials and clinical practice. In addition, the availability of long-term follow-up data from most of these trials enabled us to establish relationships of performance-test changes over 2 years with subsequent confirmed EDSS increases that further support their clinical relevance. Limitations of our work include its post hoc nature. The reported *p* values were not controlled for multiplicity and, therefore, should be interpreted with caution. In this analysis, we applied generally accepted definitions for relevant change,^21,22^ being aware that the defined steps on the ordinal EDSS and the percent change applied to define meaningful change in the performance tests represent a suboptimal consensus. The EDSS assessors were usually not directly involved in conducting the performance tests but were not formally blinded to the performance-test scores. A potential limitation of the cCDW measure proposed is that other domains of MS worsening, such as cognition,^24^ are not included. Specifically, cognitive processing speed as measured by the SDMT has been shown to add value if included in a composite score.^48,49^ Finally, in RMS trials, patients with SPMS were underrepresented by definition and comprise only a subgroup in the CONSONANCE PMS trial. However, the consistency of results across the different disease courses in the individual studies speaks for the stability and relevance of our findings.

In summary, the predictive validity of performance-test worsening as harbingers of subsequent EDSS worsening is a major finding of this study, supporting the clinical relevance of the performance tests beyond what has already been previously established, and further supports the use of cCDW/cCDP as a primary outcome measure in MS drug development programs targeting disease progression. Our findings may also lead to considering the broader use of the T25FWT and 9HPT as additional measures to more comprehensively monitor disability worsening in clinical practice because the tests are easy to perform and can be administered by trained paramedical staff.

## Glossary

9HPT: Nine-Hole Peg Test
cCDP: composite confirmed disability progression
cCDW: composite confirmed disability worsening
DBT: double-blind treatment period
DMT: disease-modifying therapy
EDSS: Expanded Disability Status Scale
FDA: US Food and Drug Administration
HR: hazard ratio
ICF: informed consent on dedicated forms
IEC: institutional ethics committees
IFN: interferon β-1a
IRB: institutional review boards
MAbs: anti-CD20 monoclonal antibodies
MS: multiple sclerosis
MSFC: Multiple Sclerosis Functional Composite
MSIS-29: 29-item Multiple Sclerosis Impact Scale
MSOAC: Multiple Sclerosis Outcome Assessments Consortium
OCR: ocrelizumab
OLE: open-label extension
PBO: placebo
PMS: progressive multiple sclerosis
PPMS: primary progressive multiple sclerosis
PRO: patient-reported outcome
RMS: relapsing multiple sclerosis
RRMS: relapsing-remitting multiple sclerosis
SDMT: Symbol Digit Modalities Test
SPMS: secondary progressive multiple sclerosis
T25FWT: Timed 25-Foot Walk Test
